# Web-Based Personalized Intervention to Improve Quality of Life and Self-Efficacy of Long-Term Breast Cancer Survivors: Study Protocol for a Randomized Controlled Trial

**DOI:** 10.3390/ijerph191912240

**Published:** 2022-09-27

**Authors:** Nelia Soto-Ruiz, Paula Escalada-Hernández, Leticia San Martín-Rodríguez, Marta Ferraz-Torres, Cristina García-Vivar

**Affiliations:** 1 Department of Health Science, Public University of Navarre, 31008 Pamplona, Spain; 2 IdiSNA, Navarra Institute for Health Research, 31008 Pamplona, Spain

**Keywords:** cancer survivors, long-term survivors, web-based intervention, quality of life, nurses

## Abstract

Long-term breast cancer survivors (>5 years free of disease) may suffer late sequelae of cancer that impact on their quality of life. The use of telehealth for cancer care is recommended but little is known about the effectiveness of digital interventions for long-term cancer survivors. This study aims to evaluate the effectiveness of a web-based personalized intervention based on artificial intelligence instead of usual primary health care to improve the quality of life of long-term survivors of breast cancer and self-efficacy for the management of late sequelae. A randomized controlled trial will be conducted. The sample will consist of long-term breast cancer survivors recruited from primary health centers. Women will be randomly assigned to the intervention group to receive a web-based personalized intervention or to the control group to receive standard primary health care by nurses. Data on quality of life of cancer survivors and self-efficacy for the management of late sequelae of cancer will be collected and assessed at preintervention, and at 3, 6, and 9 months. It is expected that, at the end of the programme, the experimental group will have improved quality of life and improved self-efficacy for the management of late sequelae of cancer.

## 1. Introduction

The cancer survivor population is growing. Early detection, therapeutic and pharmacological advances, and greater access to health services have influenced this increase in breast cancer survival [1]. The epidemiological data of the EUROCARE project on the survival of cancer patients in Europe show the change in the course of cancer, which has gone from being an acute terminal disease to a chronic process of long duration [2]. In the case of breast cancer, overall survival at 5 years after diagnosis is one of the highest in Europe [3]. The approach to cancer has also undergone significant changes in the past 25 years, achieving prolonged survival due to the improvement of treatments and care, including surveillance of recurrence and second tumors and control of symptoms and psychosocial needs. In Spain, breast cancer survival has increased from 67.5% years in 1985–1989 to 83.7% in 2010–2012, and net survival rates at 5 years are 96.6% for patients with tumors diagnosed in stage I, 88.2% for stage II, 62.5% for stage III, and 23.3% for stage IV [4].

Classically, three stages of cancer survival are distinguished [5]: acute survival (living with cancer), extended survival or intermediate survival (disease remission and monitoring of the process during the first 3–5 years), and permanent survival or long-term survival, also known as the healing and coexistence phase after cancer. Studies carried out with long-term survivors (LS) show that patients may have physical and psychosocial needs, which are often not covered by the health system [6]. Therefore, it is precisely in the stage of long survival where greater co-ordination and health continuity are needed to address the specific needs, problems, and challenges faced by LS and, in particular, long-term survivors of breast cancer (LS-BC) [7].

Currently, an area of special national and international interest focuses on the care of LS with a greater participation of primary care providers and greater continuity and co-ordination between specialized care in oncology and primary health care (PHC) [8,9]. It has been shown that shared care between oncology specialists and primary care professionals can be effective and profitable and that primary care nurses can play a relevant role in the optimization of education and promotion of health among survivors [10,11]. Likewise, patients value accessibility to health services, continuity and co-ordination of care—fundamental pillars of PHC—and show greater satisfaction when receiving individualized care focused on their needs [10].

In this context, the following care practices are recommended in the follow-up of LS-BC by primary care professionals: promotion of healthy lifestyles, personalized counselling and education for self-management of sequelae derived from treatments and care for the cancer and its symptoms (lymphedema, fatigue, pain, adverse cardiovascular events, musculoskeletal symptoms, accelerated bone loss and fractures, skin changes due to radiation, chemo brain, anxiety, depression, fear of recurrence, post-traumatic stress, altered interpersonal relationships, problems with sexuality and reproduction, altered body image, emotional, and social and work adaptation), prevention of relapses, and emotional and family support [10,12]. All of these recommendations are made with the objective of improving the living experience of the chronic process of cancer, achieving higher levels of quality of life in survivors and optimizing care resources [13,14].

However, although in the international context there are successful implementation experiences in PHC of cancer survivorship care plans [13], in Europe, and, in particular, in Spain, this implementation is moving slowly [15]. In addition, the role of the primary care team in providing care for long-term cancer survivors is not clearly defined, and few programmes have been implemented to improve care activities, standardized protocols, develop documentation and records, or update training of professionals [16,17]. Therefore, organizational and care changes are needed, favoring the implementation of structures for collaboration and shared decision making that produce greater autonomy for cancer survivors and their families.

It is key to consider the health context that surrounds us to implement health activities and programmes that are cost-effective and sustainable. The COVID−19 pandemic has led to interruptions in care, with a significant impact on the health of patients with chronic conditions or with advanced age, including breast cancer patients [18]. Therefore, it is imperative to adapt to the digital age and have innovative clinical-care information systems that, through e-health strategies, promote prevention, improve quality of life of patients, and provide individualized care to cancer patients and long-term cancer survivors [19]. Thus, information technology is a promising resource that can improve aspects, such as distance, time, and cost barriers [20], in addition to being a great advantage for reaching many patients at any time and place.

The application of information and communication technologies (ICT) in health, also known as digital health, telehealth, or e-health (used interchangeably throughout this paper), is growing at an accelerated rate. Numerous digital interventions have been carried out, as reflected in a systematic review on the subject [21]. Although there is little evidence on the benefits of these interventions for cancer survivors [22], the interventions have shown efficacy in improving patients’ psychological outlook, quality of life, and self-efficacy [21,22,23]. Considering the demonstrated effectiveness of personalized digital interventions in the acquisition of healthy habits and improvement in the quality of life, such interventions have the potential to reach a large number of users as a complement to usual health care. As a response to the health needs of a growing population, such as LS-BC in primary care consultations, it seems appropriate to propose a personalized digital intervention through the use of artificial intelligence to improve the quality of life of this population.

Therefore, the aim of this study is to evaluate the effectiveness of a web-based personalized intervention based on artificial intelligence instead of usual primary health care to improve the quality of life of long-term survivors of breast cancer (>5 years free of disease) and self-efficacy for the management of late sequelae of cancer.

This study is based on the conceptual model of quality of life in cancer survivors by Ferrell, Dow & Grant (1995) [24]. This model was developed at the City of Hope National Medical Centre in Duarte, California, and is one of the most popular in cancer survivors. The model is composed of the four domains of quality of life incorporating physical, psychological, social, and spiritual well-being [24]. Within the model, self-efficacy is instrumental for the management of cancer sequelae in cancer survivors after the medical, functional, and psychosocial consequences of cancer and its treatment.

## 2. Materials and Methods

### 2.1. Research Design

A randomized controlled trial will be conducted to respond to the following hypothesis: a personalized web-based intervention based on the prevention and management of late effects of cancer treatment will be more effective in improving quality of life of long-term breast cancer survivors and self-efficacy for the management of late sequelae of cancer by primary care providers in long-term breast cancer survivors than conventional primary health care.

Participating subjects will be randomly assigned to the 2 treatment groups: the intervention (the CUMACA-M programme) and control groups (standard care in nursing consultation). The name of CUMACA-M refers to care beyond breast cancer in its original Spanish name (CUidados Más Allá del Cáncer-Mama).

### 2.2. Methodological Framework

The study will be framed in the methodological framework of the Medical Research Council (MRC) for the development and evaluation of complex interventions [25,26]. This process has several phases, although they do not have to follow a linear sequence: Phase I modeling/design; Phase II or exploratory trial to evaluate the acceptability and feasibility of the intervention; Phase III or randomized controlled trial; and Phase IV or implementation into clinical practice. The latest update of the MRC highlights the dynamic relationship between the intervention and its context [26]. It incorporates different perspectives of intervention, efficacy, and effectiveness based on theory or systems, depending on what is already known and the additional evidence that is more useful. Four main phases of the investigation are maintained: development of the intervention or identification, feasibility, evaluation, and implementation. Each phase considers 6 basic elements: How does the intervention interact with its context? What is the underlying programme theory? How can diverse stakeholder perspectives be included in the research? What are the main uncertainties? How can the intervention be refined? Do the costs justify the effects of the intervention? These basic elements can be used to decide whether the research on a complex intervention should move to the next phase, return to a previous phase, repeat a phase, or stop.

How is complex intervention defined according to the MRC? A complex intervention generally contains several components that interact with each other and that entail a significant number of problems for the evaluators, in addition to practical and methodological difficulties. In this study, the web-based personalized programme presented below is based on a complex intervention that contains different interacting components: the type of participants, LS-BC as an emerging population; the type of intervention based on artificial intelligence; the variables analyzed that consist of several dimensions of quality of life (physical, emotional, social, and spiritual); the evaluation of the programme at 4 different times (preintervention, 3 months, 6 months, and 9 months); and the specific context of primary health care. All these components make the intervention of this protocol complex and, therefore, suitable for the MRC framework. In addition, it is a digital health intervention and a web-based tailored intervention, and most web-based interventions are complex interventions [27].

**Figure 1:**
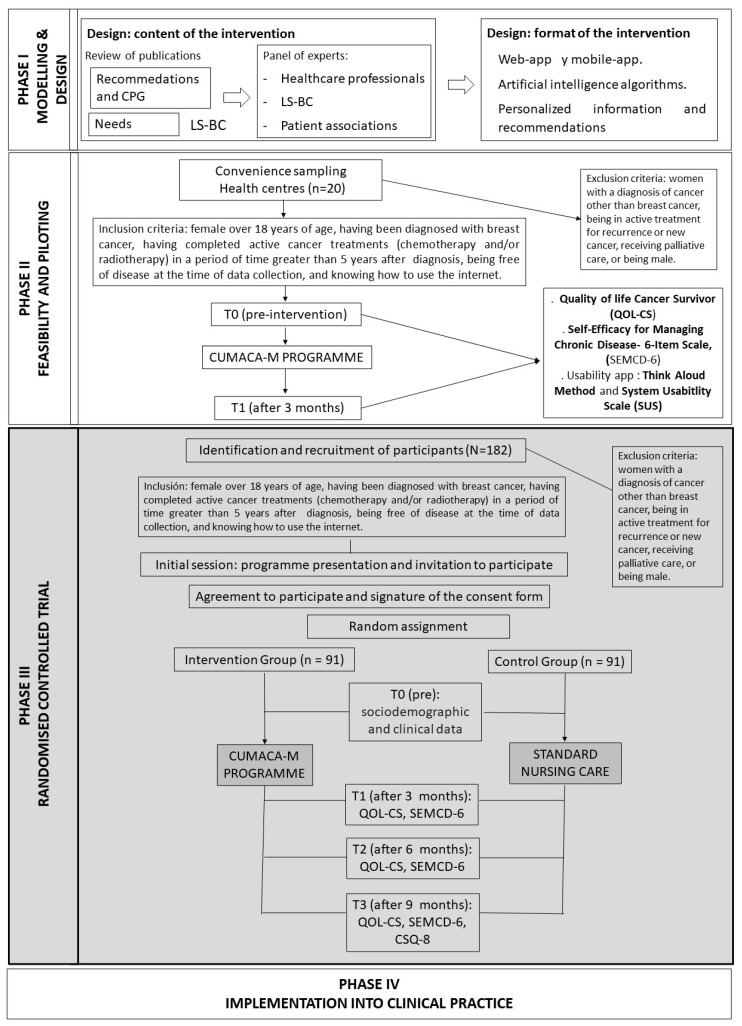
Phases of the study.

The protocol presented here focuses on Phase III related to the randomized controlled trial (Figure 1, grey colour). Phase I of the intervention is being developed through the review of publications on recommendations and clinical practice guidelines regarding breast cancer survivors, their health care needs once the treatments have been completed, in addition to guidance from a panel of experts including health care professionals from specialized care and PHC (oncologists, cancer nurses, psycho-oncologists, general practitioners, and community nurses), LS-BC and breast cancer patient associations. The result will allow configuration of the content of the intervention. Simultaneously, in this same phase, the digital health intervention will be designed (web and mobile application format). Once this evidence is available, in Phase II of the MRC, the intervention will be piloted with LS-BC (*n* = 20) to evaluate the feasibility of the intervention protocol, to know the usability of the web programme and to detect possible risks, failures, or problems and to solve them before completing the study in Phase III.

In Phase IV, considering the current situation and implementation in Spain of the Comprehensive Care Plan for Long-term Cancer Survivors of the Spanish Society of Medical Oncology [5], the intervention will be negotiated with the competent health authorities of the region for its implementation in the region’s primary care centers. An innovative practice in the provision of health services, which will make it possible to remove barriers due to distance, time, and cost, in addition to reaching many patients simultaneously at any time and place. In turn, this will allow personalized care for long-term cancer survivors.

### 2.3. Settings

The study will be carried out in Navarra, an autonomous community and province in northern Spain, with a total population of 482,916 people. Six primary health centers from different municipalities in northern (country blinded for review) will be selected to participate in the research. If the sample size of long-term cancer survivors is not reached, new primary health centers will be incorporated.

### 2.4. Inclusion and Exclusion Criteria

The subjects will be LS-BC who have completed active treatments and who meet the following inclusion criteria: female over 18 years of age, having been diagnosed with breast cancer, having completed active cancer treatments (chemotherapy and/or radiotherapy) in a period of time greater than 5 years after diagnosis, being free of disease at the time of data collection, and knowing how to use the internet. Exclusion criteria are as follows: women with a diagnosis of cancer other than breast cancer, being in active treatment for recurrence or new cancer, receiving palliative care, or being male. Although almost 1% of breast cancer diagnoses are in men, they will not be included in this study because their needs may be different from those of women, especially in relation to the psychosocial dimension.

### 2.5. Sample Size

For the calculation of the sample size, a primary outcome measure, quality of life (QOL), was selected [28]. The QOL measure used for this study goes from 1 (extremely poor) to 10 (excellent). A Cohen’s d of 0.4 (effect size) was established, taking into account a difference in quality of life of 0.75 and a standard deviation of 1.8. This effect size has been established, taking into account previous studies [29], and is expected to occur over time, after the intervention. For its part, the type I error has been set at 5% and the type II error at 20%. According to these parameters, 91 subjects will be needed in the intervention group (IG) and 91 subjects in the control group (CG). Depending on the proportion of losses that may occur, the sample size will be increased, calculating an adjusted sample size. In the event that the pilot study suggests making modifications in the calculation of the sample size due to a re-estimation of the effect size, the appropriate corrections will be made before beginning the study. In this study, sample size estimation was performed using the Granmo Calculator, available online in several languages (https://www.imim.cat/ofertadeserveis/software-public/granmo/) (accessed on 15 May 2022.).

### 2.6. Randomization and Allocation Concealment

The randomization will be by blocks, and this will be divided by the number of subjects in each block (health center). Six primary health centers will participate, so that, in each block/health center, the sample will be 32 subjects (16 IG and 16 CG). This would be a total of 192 participants, which would cover the calculated sample size of 182 subjects).

A list of random numbers generated by the EpiInfo 7.1 programme will be used to randomly assign participants to groups and to minimize the variation in external factors that can affect the comparison. The randomization method will be masked; therefore, the researchers who perform the randomization will not know to which group each subject will be assigned. To mask the randomization process, sealed and opaque envelopes will be sequentially numbered.

### 2.7. Description of the CUMACA-M Intervention

The personalized digital intervention will be designed in web and mobile application format to facilitate access at any time and from any place, regardless of the device used (computer, tablet, or smartphone). As it is a personalized intervention with the use of different algorithms using artificial intelligence, participants will receive information adapted to their personal needs. As mentioned above, the contents will be developed from an exhaustive review of recommendations and clinical practice guidelines for the care of LS-BC and on the main needs of LS-BC (Phase I of the MRC). Subsequently, the content of the intervention will be agreed upon by a panel of multiprofessional experts from PHC and specialized care, as well as an LS-BC and a representative of an association supporting women with breast cancer.

These contents will be configured in different modules according to the areas identified as priority needs of the LS-BC (for example, fatigue module, lymphedema module, work activity module, and fear of recurrence module), and the principles of the educational intervention characteristic of PHC consultations will be followed (feedback methods, risk personalization, increased awareness, active learning, persuasive communication, goal setting, and actions) [30].

The main page of the digital intervention programme will show a personal record and a video on how to use the programme. The intervention will begin with a screening questionnaire that will measure several concepts that impact quality of life, such as fatigue, anxiety, fear of recurrence, or return to work. According to the responses, participants will receive personalized advice by means of artificial intelligence algorithms on which modules deserve more attention. The web application itself will integrate the measurement of outcome variables that will allow evaluation of the effectiveness of the intervention. The effective management of the topics identified for the intervention will ultimately improve the quality of life of the LS-BC.

In Phase II (feasibility and piloting), the pilot of the intervention will be carried out through a pre-experimental pretest–post-test design with a single group that will pilot the intervention and test the usability of the application to identify areas for improvement and introduce timely modifications.

### 2.8. Evaluation Measures

The independent variable will be the CUMACA-M programme, since it is expected that, after its application, there will be changes in the values of the dependent variables (see below). Measures of the variables of interest will be collected, both in the IG and in the CG, at the preintervention time (T0), at 3 months after the intervention (T1), at 6 months (T2), and at 9 months (T3).

The dependent variables analyzed will be quality of life (QOL) of cancer survivors (CS) and self-efficacy in the management of cancer sequelae.

Quality of Life—Cancer Survivors (QOL-CS scale, Spanish version): it will be used with cancer survivors in QOL [24] and its 41 items in the 4 domains of QOL (physical, psychological, social, and spiritual well-being) will be measured on a Likert-type scale from 1 (extremely poor) to 10 (excellent). The overall QOL-CS tool test–retest reliability was 0.89, with subscales of physical 0.88, psychological 0.88, social 0.81, and spiritual 0.90 [24]. Cronbach’s alpha coefficient is a measure of agreement between items and subscales. The analysis revealed an overall r = 0.93. Subscale alphas ranged from r = 0.71 for spiritual well-being, r = 0.77 for physical, r = 0.81 for social, and r = 0.89 for psychological. Cronbach’s alpha is 0.93. Subscale alphas ranged from 0.71 for spiritual well-being, 0.77 for physical, 0.81 for social, and 0.89 for psychological [24].

Self-Efficacy for Managing Chronic Disease 6-Item Scale (SEMCD−6, Spanish version): it will be used to assess self-efficacy in the management of cancer sequelae and promotion of healthy lifestyles [31]. It is structured into 6 items related to the control of symptoms, role performance, emotional state, and communication with health professionals. In this study, the Spanish version of the SEMCD will be used that is structured into 4 items compared to the 6 in the English version [31].

The sociodemographic data of the LS-BC (age, sex, marital status, educational level, and employment status) will be collected through an ad hoc questionnaire at the beginning of the intervention. Data on the medical history (type of breast cancer, type of treatments, time elapsed since the end of active cancer treatment, relapses, and comorbidities) and follow-up will be collected through the clinical history of the LS-BC.

At the end of the intervention, satisfaction with the care received (CUMACA-M programme for the IG and standard nursing care for the CG) will be measured through the Spanish version of the CSQ−8 Satisfaction Questionnaire [32]. Each of the 8 items has 5 response options (from very dissatisfied to very satisfied).

### 2.9. Data Collection

The intervention group protocol is as follows: the CUMACA-M programme is based on a web application for long-term breast cancer survivors. Participants (LS-BC) in the IG will receive the personalized digital intervention (CUMACA-M programme). Coinciding with the initial session of acceptance to participate in the project and signing a consent form, after the random assignment to the IG, the web application will be presented. Registration will be performed (with username and password), and instructions for use will be provided. At that time, preintervention and sociodemographic data will be collected, as well as measurements of QOL and self-efficacy in the management of the disease. Medical data will be collected in an electronic questionnaire with the help of the SurveyMonkey^®^ tool. For this, a duration of one hour in a face-to-face session is estimated. Subsequent measurements of QOL and self-efficacy variables will be performed at T1, T2, and T3, from the beginning of the intervention. Through the web application, a message will be sent to participants prompting them to complete the questionnaire. This reminder will be reinforced by email and through a WhatsApp^®^ distribution list (differentiated lists for LS-BC of the IG and CG). Satisfaction data with the web application will be collected only at 9 months (T3).

The control group protocol consists of standard care in the PHC nursing office. Since there are no defined nursing care procedures for long-term cancer survivors in PHC in Spain, the LS-BC in the CG will be limited to a single face-to-face consultation of an estimated duration of 1 h, in which participants will receive an informative brochure on topics related to life after cancer and will take the opportunity to offer brief advice on healthy lifestyle habits regarding the importance of a balanced diet and regular exercise. In this session, information about the study will also be provided and, after acceptance to participate, information about the variables to be collected over time (T0, T1, T2, and T3) will be explained. The sociodemographic and medical data of the LS-BC will be collected in T0, as well as the data for the QOL and self-efficacy variables in the management of the disease. An electronic questionnaire will be designed with the help of the SurveyMonkey^®^ (Momentive, San Mateo, CA, USA, 2022) tool to be completed during the consultation on a tablet. Subsequently, participants will be contacted by email and a WhatsApp^®^ (2.22.20.75, Meta Platforms, Menlo Park, CA, USA, 2022)) distribution list with an invitation to complete a questionnaire that will evaluate their QOL and self-efficacy at T1, T2, and T3. Satisfaction data with the care received (standard nursing care) will be collected at 9 months (T3).

### 2.10. Data Analysis

The statistical analysis of the data will consist of a descriptive presentation of the parameters analyzed in the CUMACA-M programme. Continuous variables will be described as the mean and standard deviation. Categorical variables will be presented as frequencies and percentages. Subsequently, after assessing the normality of the data, corresponding inferential analyses will be performed. During the first stage, an assessment of the homogeneity of the groups (IG and CG) will be carried out by comparing the outcome variables (QOL and self-efficacy), sociodemographic variables, and clinical variables. For this, Student’s t test will be used for the quantitative variables, the chi-square test for the nominal or dichotomous variables (with the corresponding Fisher’s correction, if necessary), and the Wilcoxon test for variables of an ordinal nature. In a second stage, the differences in the outcome variables between the 2 groups (IG and CG) over time (T0, T1, T2, and T3) will be analyzed using multivariate analysis (MANOVA), in which the “group” factors will be incorporated and “time”, as well as the interaction between the 2. In all cases, the significance level will be set at 0.05. The data will be analyzed with the help of SPSS 24.0 software (IBM, Chicago, IL, USA, 2022)).

The intervention will be considered effective if the two variables evaluated (quality of life and self-efficacy) show results of improvement in the intervention group compared to the control group.

### 2.11. Validity and Reliability/Rigour

This study is part of the Medical Research Council framework for the development and evaluation of randomized controlled trials for complex interventions in health. The invitation to participate in the study will always be made by the same researchers and, once the consent is signed, the random assignment will be made to the CG or the IG. Similarly, the initial session in both groups will be conducted by trained nurses who are knowledgeable about the research protocol.

As informed earlier, the instruments that will be used for data collection (QOL-CS and SEMCD) are validated and translated into Spanish and are the most widely used for the targeted population group.

In the IG, the questionnaires will be integrated into the CUMACA-M programme itself and, in the CG, an online electronic tool will be used for their collection. The research consists of a plan for the management and treatment of participants’ data, in which the method of data collection, its custody, and the restriction of access to authorized research team personnel is indicated. This plan was developed with advice from the Data Protection Officer of the Public University of Navarra.

### 2.12. Ethical Considerations

This project has been approved by the Clinical Research Ethics Committee of Navarra (PI_2021/18), and the necessary institutional authorization will be obtained to carry out the study. All participants in Phases II and III of the study will receive an information sheet about the research, their degree of involvement, the treatment of the data, and the legal requirements. Likewise, they will receive, in paper format, the informed consent (shown as supplementary materials) that they must sign if they agree to participate, voluntarily, in the research. Furthermore, participants will be informed that they can leave the study at any time without having to justify themselves and will be informed of the reference person for any problem or doubt related to the project. If participants in any of the groups have any questions or become distressed for any reason and need extra support, they will be able to contact a reference person through a telephone number that will be provided at the beginning of the study (included in the information sheet of the research). The study will have exhaustive compliance with all ethical-legal requirements in force related to research with humans. In the event that the CUMACA-M programme is shown to be effective in improving QOL and self-efficacy in LS-BC, after the investigation, the CG will be invited to obtain a username and password to access the programme for 9 months (See Supplementary Materials for information sheet form for participants.)

## 3. Discussion

We describe the development and piloting plan for evaluating the effectiveness of a web-based personalized intervention, based on artificial intelligence, a novel intervention to improve the quality of life of free-of-disease breast cancer survivors and self-efficacy for the management of late sequelae of cancer in the context of primary health care. We believe this research is of interest, as the number of people in Navarra who survive breast cancer after five years post-diagnosis is growing significantly [4]. Some of these LS-BC are reincorporated into daily life without a problem; however, on many occasions, these women experience consequences: physical (chronic fatigue, pain, etc.), psychological (anxiety, depression, and fear of relapse), social (altered interpersonal relationships and difficulty in returning to work), and spiritual [10,12]. These sequelae or health problems, for many, are not addressed by health professionals [21]. Therefore, the need to promote greater continuity and co-ordination between specialized care in oncology and PHC that favors the specific monitoring of these women in the community is crucial.

Web-based personalized interventions make access possible at any time and place through a mobile device. In addition, they make it possible to individualize interventions with the help of artificial intelligence algorithms tailored to the specific needs of each patient. Personalization can be static, with a single initial evaluation that guides the programme or dynamic, with multiple evaluations that initiate the evolution of the intervention [23]. While it is true that studies have shown a clear commitment in favor of static or dynamic interventions, the effectiveness of these interventions also depends on the variables that are selected for personalization (e.g., motivation and trust), the resources that are used (videos, email, etc.), the level of intensity of the intervention, and the characteristics of the targeted population [22]. Personalization is produced from the initial evaluation of the characteristics of the individual and in relation to the objective of interest so that information or content relevant to them is provided according to their particular needs [33]. The CUMACA-M programmme of our research will aim to respond to the specific needs of each breast cancer survivor, offering personalized care as nurses do with their patients in the primary care setting.

There is evidence of the efficacy of digital intervention in studies on behavioural changes, both in the short term and long term; the effects are significantly greater compared to other types of nonpersonalized interventions in terms of the acquisition of healthy behaviors, such as physical activity, healthy eating, and smoking cessation, and improvements in quality of life [33]. In patients with cancer, successful experiences have also been seen; in the Netherlands, for example, a personalized digital intervention provides psychosocial support and promotes healthy lifestyles in patients who have completed primary treatments (surgery, chemotherapy and/or radiotherapy) for 1 and 12 months after diagnosis [34]. Therefore, the use of telehealth in cancer patients is recommended. However, little is known about the effectiveness of web-based personalized interventions through artificial intelligence for long-term cancer survivors. Therefore, this study will meet a gap in the literature by evaluating the effectiveness of a web-based tailored intervention to improve the quality of life of long-term survivors of breast cancer in the context of primary health care.

Although we anticipate that the CUMACA-M intervention will present a novel intervention for long-term cancer survivors, limitations of the study should be considered. One of the limitations of this study is that only women who have experienced negative sequelae (such as chronic fatigue, lymphedema, etc.) following breast cancer treatment may volunteer for this study. Furthermore, it could be that older survivors have less computer knowledge and use the internet less as a source of health-related information than younger survivors. Therefore, it is intended to develop a user-friendly and easy-to-use digital programme, including a quick user guide and an explanatory video on the operation of the digital programme. Likewise, although the participants of both groups will be asked not to use other mobile applications to improve their physical activity, we cannot guarantee their compliance.

## 4. Conclusions

The implementation of a personalized digital intervention will have a direct impact on women who have had breast cancer for years and may have specific physical, psychological, social, and work issues that need to be addressed by health professionals in the community. Once it is known if this intervention is effective in improving and promoting health in LS-BC, the integration of the intervention in primary health care of the region where the study is carried out is expected to cover the need for follow-up of the LS-BC and promote the continuity of care in the different stages of cancer. Thus, the innovative digital intervention can be an effective tool that allows primary care providers to address the needs of long-term cancer survivors.

## Data Availability

Not applicable.

## Supplementary Materials

The following supporting information can be downloaded at: https://www.mdpi.com/article/10.3390/ijerph191912240/s1; Patient consent informed.
