# Supplementary material for: Web-Based Personalized Intervention to Improve Quality of Life and Self-Efficacy of Long-Term Breast Cancer Survivors: Study Protocol for a Randomized Controlled Trial
Source: Int J Environ Res Public Health. 2022 Sep 27;19(19):12240. doi: 10.3390/ijerph191912240 (PMC9564785; doi:10.3390/ijerph191912240)
Supplement: Supplementary file 1 [file ijerph-19-12240-s001.zip › ijerph-1889892-supplementary.pdf]

Information Sheet for participant - CUMACA-M Project

Dear patient,

We are a group of nurse researchers, professors of the Department of Health Sciences of the Public University of Navarre (UPNA), and members of the Inno-CARE Research Group.

We are writing to invite you to participate in the CUMACA-M Project "Care Beyond Breast Cancer". The aim of this study is to design, implement and evaluate a web-based personalised program based on artificial intelligence, to improve the quality of life of long-term breast cancer survivors and their self-efficacy for the management of late sequelae of cancer and treatments.

The participants will be randomly distributed into two groups (A and B) without knowing the group to which they will be assigned. The participants in group A will receive the usual primary health care. The participants in group B will have access to a new web/mobile application with personalized content related to health and wellness after finishing cancer treatments. When the study is completed, participants in group A will have the possibility of accessing the web/mobile application with personalized content.

For all the participants in the study, participation implies your commitment to fill in an electronic questionnaire with questions related to your quality of life and disease management on four occasions (at the beginning of the study, and 3, 6 and 9 months after the study initiation). Acceptance of participation also implies granting permission to the research team to access data from your medical record, including sociodemographic data (age, sex, marital status, educational level, and employment status) and clinical data (type of breast cancer, type of treatment(s) received, time since the end of active cancer treatment, cancer-related-problems, and comorbidities).

Confidentiality of your data will be always ensured during the study. The personal data that is required are those necessary to cover the aim of the study. Your name will not appear in any of the project reports, and your identity will not be revealed to anyone except for the purposes of the study. The handling of the data will be carried out in encrypted form, ensuring their anonymity and any information of a personal nature that may be identifiable will be kept and processed in secure conditions, being restricted to authorized personnel who will be obliged to maintain the confidentiality of the information. The results of the

study may be communicated to the health authorities and, eventually, to the scientific community through conferences and/or publications.

In accordance with current law, you have the right to access your personal data. Likewise, and if justified, you have the right to its rectification and cancellation. If you wish, you could request it from the researcher who assists you in this study.

RISKS OF RESEARCH FOR THE PATIENT: NONE

Participation does not imply any risk and, on the other hand, can bring you notable benefits related to a greater knowledge of the management of the sequelae of breast cancer and an improvement in your quality of life.

IMPLICATIONS FOR THE PATIENT:

- Participation is completely voluntary.
- The patient can withdraw from the study at any time, without giving explanations and without this affecting his/her care and health care.
- The information obtained will be used exclusively for the specific purpose of this study.
- The non-acceptance of participation in this study will not condition in any way the care received.

If you require additional information, you can contact our research staff by phone: xxxxxx or by email: xxxxxx

Thank you very much for your help.
